# Rapid Ventilator Splitting During COVID-19 Pandemic Using 3D Printed Devices and Numerical Modeling of 200 Million Patient Specific Air Flow Scenarios

**DOI:** 10.21203/rs.3.rs-48165/v1

**Published:** 2020-08-12

**Authors:** Muath Bishawi, Michael Kaplan, Simbarashe Chidyagwai, Jhaymie Cappiello, Anne Cherry, David MacLeod, Ken Gall, Nathan Evans, Michael Kim, Rajib Shaha, John Whittle, Melanie Hollidge, George Truskey, Amanda Randles

**Affiliations:** 1 Department of Surgery, Duke University Hospital; 2 Department of Biomedical Engineering, Pratt School of Engineering, Duke University; 3 Department of Respiratory Therapy, Duke University Hospital; 4 Department of Anesthesiology, Duke University Hospital; 5 Department of Mechanical Engineering, Pratt School of Engineering, Duke University; 6 restor3d Inc, Durham NC

## Abstract

There has been a pressing need for an expansion of the ventilator capacity in response to the recent COVID19 pandemic. To address this need, we present a system to enable rapid and efficacious splitting between two or more patients with varying lung compliances and tidal volume requirements. Reserved for dire situations, ventilator splitting is complex, and has been limited to patients with similar pulmonary compliances and tidal volume requirements. Here, we report a 3D printed ventilator splitter and resistor system (VSRS) that uses interchangeable airflow resistors to deliver optimal tidal volumes to patients with differing respiratory physiologies, thereby expanding the applicability of ventilator splitting to a larger patient pool. We demonstrate the capability of the VSRS using benchtop test lungs and standard-of-care ventilators, which produced data used to validate a complementary, patient-specific airflow computational model. The computational model allows clinicians to rapidly select optimal resistor sizes and predict delivered pressures and tidal volumes on-demand from different patient characteristics and ventilator settings. Due to the inherent need for rapid deployment, all simulations for the wide range of clinically-relevant patient characteristics and ventilator settings were pre-computed and compiled into an easy to use mobile app. As a result, over 200 million individual computational simulations were performed to maximize the number of scenarios for which the VSRS can provide assistance. The VSRS will help address the pressing need for increased ventilator capacity by allowing ventilator splitting to be used with patients with differing pulmonary physiologies and respiratory requirements, which will be particularly useful for developing countries and rural communities with a limited ventilator supply.

## Introduction

The COVID19 pandemic has shed light on the need for emergency ventilator systems which can be rapidly deployed when the demand for ventilators surpasses their supply[1], such as during regional emergencies[2], global pandemics[3], and in low-resource ICUs[4]. These various scenarios require a ventilator sharing strategy that maximizes the number of patients able to receive potentially life-saving treatment from a limited number of ventilators. Ventilator splitting has been introduced as a strategy to support multiple patients on the same ventilator and has been implemented at a number of institutions during dire situations[5]–[7]. Recent advances, such as the addition of resistors[8], clamps[9], and valves[10], has allowed ventilator splitting to be useful for carefully matched patients[7]. However, ventilator splitting remains unable to be safely and rapidly implemented for patients with significantly differing pulmonary compliances[9] or minute ventilation requirements[10], as this could lead to volutrauma, barotrauma, and/or hypoventilation of one or both of the patients. Concerns related to the safety of ventilator splitting has prevented it from being recommended as a general solution for ventilator shortages in the most extreme of circumstances[11].

To address this problem, our group developed a rapidly deployable and low-cost ventilator splitter and resistor system (VSRS) with 3D-printed interchangeable airflow resistors coupled with numerical modeling to allow for patients with differing pulmonary mechanics to share the same ventilator. The resistors allow independent differential control over the tidal volumes and pressures delivered to each patient and a computational model was created to quantify how ventilator settings, endotracheal tube diameters, and patient pulmonary compliances affect delivered tidal volumes and pressures in the VSRS. When used together, the 3D printed components and the numerical modeling allow clinicians to quickly, but safely ventilate multiple patients, even when the patients have differing ventilatory requirements and pulmonary compliances.

In this work we described the (a) development of the novel VSRS with customizable, 3D printed resistors, (b) validation of the system against benchtop data, (c) development and validation of a predictive, personalized computational model, and (d) use of a massively parallel computing framework to capture the widest possible expanse of clinically relevant scenarios. This system and the decision support application is currently under review for FDA EUA approval.

## Results

### Rapid point of care 3-D printing manufacturing of the VSRS system

The VSRS consists of two primary components: the splitter and the resistor. The splitter component is a Y-shaped adaptor that splits a single airflow into two separate channels (used for the inspiratory limb splitting). When used in reverse, the splitter can combine airflow from two channels into a single channel (used for the expiratory limb). The splitter has a continuously graded diameter such that the interior diameter of the splitter’s single-channel end fits over the exterior diameter of standard ventilator tubing, while the exterior diameters of the splitter’s dual-channel ends fit within the interior diameter of standard ventilator tubing. The splitter features a 60° junction between the two dual-channel ends. The splitter fits standard ventilator tubing and at least two splitters would be required within a shared ventilator circuit. The resistor is an inline adaptor that incorporates a continuously narrowing lumen to provide increased resistance to airflow relative to the airflow through the splitter or standard ventilator tubing. Different sized resistors were created and tested. All resistors have identical exterior dimensions, the only differences being the final lumen diameter and the continuously graded reduction in lumen diameter. The resistor would be installed within a ventilator circuit upstream of a single patient to control airflow to that patient at a clinician’s discretion. Resistors are clearly labeled with the reduced lumen size on their input end. The interior diameter of the input end fits over the exterior diameter of standard ventilator tubing, while the exterior diameter of the output end fits within the interior diameter of standard ventilator tubing. This design ensures resistors can only be incorporated into a ventilator circuit in one orientation.

The components of the VSRS are manufactured from a commercially available, photopolymer via stereolithography (SLA), using a commercially available printer (FormLabs 2 or 3, Formlabs Inc, Somerville, MA). Both components are fully solid, continuous parts. The photopolymer is marketed as “Durable Resin” by Formlabs Inc. (Somerville, MA). When used to manufacture parts via SLA, Durable Resin produces parts that are frosted clear with mechanical properties similar to those of polypropylene. The quality systems for medical grade printing is provided by restor3d Inc. (Durham NC).

As part of the FDA EUA application, the material strength and durability of the printed VSRS system were tested and compared to standard ventilator parts/tubing. They were found to match or exceed those of commercially available ventilator tubing connectors (data not show). Furthermore, per FDA guidance, the VSRS system was tested (by a third independent party) for volatile organic compounds and particulate matter sampling and found to not pose any significant biological risk evaluated by an independent toxicologists.

### Design of the VSRS and benchtop circuit

Overall design of the VSRS can be seen in Figure 1A and 1B, with the benchtop circuit displayed in Figure 1C. The circuit incorporated a number of one-way valves at the inspiratory and expiratory limbs to limit mixing and viral/bacterial filters were placed at locations of possible cross contamination. Using ventilator settings with a respiratory rate of 20 bpm (breaths per minute), a PEEP (positive end-expiratory pressure) of 5 cmH_2_O, and PIP (peak inspiratory pressure) of 20 cmH_2_O, the delivered tidal volumes to the low compliance artificial lung was 352–359 ml and 566–567 ml to the medium compliance lung (Table 1). The precise compliance of the test lungs in ‘low’ configuration was determined to be 18 ml/cmH_2_O, and 34–36 ml/cmH_2_O for the ‘medium’ configuration (Table 1).

### Ventilator splitting with differing test lung compliances results in significantly different tidal volumes

As expected, splitting the ventilator across two test lungs when their compliances differed resulted in significant differences in delivered tidal volumes to each lung (Figure 2). At a respiratory rate of 15, the medium compliance lung received on average ~60% more tidal volume than the low compliance lung (Figure 2). The delivered tidal volume was similar when both lungs had either low or medium compliance. Respiratory rate had an inverse effect on differential tidal volume delivery to lungs with different compliances, with increasing respiratory rates resulting in decreased differential flows (Figure 2).

### The 3D printed resistor can normalize airflow to lungs with differing compliances

The effect of the 3D printed resistor on tidal volumes was studied by adding resistors of varying radii to the splitter of the aforementioned circuit. All experiments demonstrated a decrease in delivered tidal volumes to the limb with the resistor, with a greater decrease occurring for smaller resistor apertures (Figure 3). Consequently, when placed on the limb of the circuit going to the greater compliance lung, the resistor acts to decrease the differential delivered tidal volume (Figure 3). For the precise ventilator settings, endotracheal tube sizes, and pulmonary compliances used in Figure 4, the 3.5 mm resistor lead to fully matched tidal volumes to both lungs. A smaller resistor radius of 3.0 mm resulted in the low compliance lung actually receiving a larger tidal volume than the higher compliance lung (Figure 3). These experiments emphasize how small changes in resistor size can lead to large changes in delivered tidal volumes.

### The computational model predicts benchtop measurements

The computational model was validated against the test lung data. There is excellent agreement between the two, including agreement when different resistor sizes are used (Figure 4), which is evidenced by a Pearson correlation coefficient of 0.9697 and a p-value of less than 0.0001. Both the benchtop data and the computational model agree that a 3.5 mm resistor on the higher compliance limb will result in equivalent delivered tidal volumes to both lungs for this specific configuration (Figure 4).

More generally, the computational model supports the findings from the benchtop model that adding a resistor to the split ventilator circuit significantly alters delivered tidal volumes and pressures. Figure 5 illustrates the model outputs of predicted pressure, flow rate, and volume waveforms for a patient with a lower pulmonary compliance (Patient A) sharing a ventilator with a patient with higher pulmonary compliance and a resistor (Patient B). The characteristic waveform of pressure-controlled ventilation is observed for patient A, where the ventilator reaches the ventilator-specified peak inspiratory pressure and then a plateau occurs. However, for patient B, the resistor slows the buildup of pressure and the ventilator-specified peak inspiratory pressure is never reached. As a result, the flow rate waveform for patients A and B markedly differ. Patient A has decreasing flow rates during the inspiratory pressure plateau due to the increased resistive force of the lungs as they continue to distend under constant pressure. However, the pressures for Patient B continue to increase throughout inspiration and therefore flow rates do not decrease as inspiration continues, resulting in a flow rate waveform which more closely resembles volume-controlled ventilation. This differential behavior of flow rates throughout inspiration results in a high sensitivity of differential tidal volumes to respiratory rate when using a resistor, which was also observed in the benchtop experiments (Figure 2).

### Pressure-controlled ventilation protects patients from changes in the opposing patients circuit

The computational model allowed for the exploration of both pressure-controlled and volume-controlled ventilator splitting. Figure 6 illustrates how in pressure-controlled ventilation both the tidal volumes and pressures to patient A are not affected by changes to patient B’s circuit, such as the addition of a resistor, which was a trend also observed in the benchtop data (Figure 3). In both pressure-controlled and volume-controlled ventilation, the pressure delivered to the bifurcation at the circuit is always equal to each branch, and therefore the delivered tidal volumes to each patient are independently a function of how their pulmonary characteristics respond to this pressure. However, in volume-controlled ventilation, a coupling of the delivered tidal volumes between the two patients occurs. When the resistance of one limb of the circuit increases, the ventilator senses the resulting decrease in combined delivered tidal volume and subsequently increases the delivered pressures in an attempt to achieve the desired tidal volume, which results in increased pressures and volumes to both patients. This differs from pressure-controlled ventilation, where patient A is protected from experiencing dangerously elevated pressures and tidal volumes in response to changes in resistance in patient B’s circuit (Figure 6). Therefore pressure-controlled ventilator splitting offers a markedly improved safety profile due to a reduced risk of barotrauma or volutrauma to one patient from changes in the opposite patient’s circuit.

### Tidal volumes from pressure-controlled ventilation are highly sensitive to small changes in ventilator settings, endotracheal tube sizes, and patient characteristics

Predicted tidal volumes from pressure-controlled ventilation are sensitive to small changes in multiple parameters (Table 2) in a non-linear fashion (Figure 7). At lower compliance values, different endotracheal tube sizes result in only minimal changes in tidal volumes and tidal volumes increase roughly linearly with compliance. However, as Figure 7 illustrates, for larger compliance values there is a marked change in the increase in tidal volume as a function of pulmonary compliance and significant differences in tidal volumes due to different endotracheal tube sizes can occur. This is an important characteristic to consider as pulmonary compliance is expected to change due to disease progression or recovery. For example, safe tidal volumes for a given set of patient characteristics and ventilator settings while the patient has low pulmonary compliance would become dangerously high as the patient improves, and therefore a different resistor size needs to be used. Figure 7 also demonstrates the non-linear effect of respiratory rate (RR) on tidal volumes, where the sensitivity of tidal volumes to changes in RR is much greater for lower RRs than for higher RRs. These and other non-linearities necessitate a high-resolution parameter exploration in order to safely and precisely quantify the effect of multiple patient parameters and ventilator settings on delivered tidal volumes.

### The computational model solves the large parameter space of potential patient pairings

To properly account for the range of different factors influencing patient-delivered tidal volumes in a split-ventilator configuration, we performed the largest-to-date computational effort to simulate the necessary number of different ventilator settings and patient-specific parameters that clinicians may encounter. Figure 8 displays the results of exploring the seven-dimensional parameter space (Table 2) which were found to significantly affect predicted tidal volumes and pressures. Over 200 million different simulations were required to explore the parameter space at a sufficient resolution such that the step size for a given parameter resulted in less than a 5% change in tidal volume.

Figure 8 depicts the scale of the parameter sweep that was performed. The upper left panel of Figure 8 shows how increasing the driving pressure (PIP-PEEP), as well as increasing the I:E and therefore the inspiratory time, acts to increase tidal volumes. The upper right panel of Figure 8 is an expansion of the predicted tidal volumes of the upper left panel by selecting a PIP of 28, PEEP of 8, and I:E of 1 and illustrating tidal volumes as a function of endotracheal tube sizes, RR, and compliance. The interplay of multiple parameters is observed as the effect of changing RR on tidal volumes is itself dependent on the patient’s pulmonary compliance and endotracheal tube size. Lastly, by selecting a specific pair of patient’s pulmonary compliances and endotracheal tube sizes, the bottom panel of Figure 8 depicts how the resistor can finely control delivered tidal volumes and maximum pressures. Larger resistor sizes approach the tidal volumes and pressures for the cases without a resistor and differences in tidal volumes and pressures due to a 0.5 mm change in resistor size decrease as the resistor size increases.

Using this data, a mobile application was developed to provide decision support for clinicians to determine the optimal resistor size to implement for a given patient pairing. The app allows the clinician to enter the relevant ventilator settings (peak inspiratory pressure, positive end expiratory pressure, inspiratory to expiratory ratio, and respiratory rate), patient-specific pulmonary compliances, and endotracheal tube diameters (Table 2). The app then displays the predicted delivered tidal volumes and maximum pressures to each patient during a ventilator split for each possible different resistor size.

## Discussion

COVID-19 has brought renewed interest[12] and innovation[10], [13] in ventilator sharing, which has applications in future respiratory outbreaks, the battlefield, and in low resource ICU settings, as well as during the current global pandemic. However, current advances in ventilator splitting still require careful patient matching[14] or the use of complex medical equipment[10] that might not be feasible in all cases. The VSRS system implements simple 3D printed components that can be readily created in locations with a 3D printer and shipped to nearby hospitals, as well as a free mobile app that removes the guess work from pairing patients and determining what resistor size to use.

In the first part of this work, we demonstrate the large difference in delivered airflow to two lungs of different compliances sharing the same ventilator. This confirms the feared clinical scenario that would lead to one patient potentially experiencing volutrauma and/or the other having inadequate ventilation. Using 3D printed airflow resistors in the circuit for the patient with the higher compliance lungs allows for control over the delivered tidal volume to the higher compliance lung. The airflow can be predicted using the computational model we have developed, which allows clinicians to select the resistor that will result in the desired tidal volumes for each patient, even for patients with very different ventilation needs and pulmonary compliances. A mobile app has been created to assist clinicians with this process.

Shared ventilatory support still poses significant risk of harm and should not be undertaken unless there are no other viable options. An important safety concern with ventilator splitting relates to viral/bacterial cross contamination. Our circuit setup uses strategically placed viral/bacterial filters and one-way valves to decrease the chance of cross contamination. Additional safety measures included the use of EtCO2 monitors for each patient, frequent blood gas measurements, and clinical examination to rapidly diagnose problems and correct them.

The numerical model helped to elucidate the dynamics behind the superior safety profile of pressure-controlled ventilation over volume-controlled for patients sharing a ventilator. Modeling and benchtop data found that respiratory rate plays an important role on differential tidal volumes during ventilator splitting to two patients with different compliances. As the respiratory rate increases, differential airflow to the different compliance lungs decreased, with an overall drop in delivered tidal volumes, but not minute ventilation, to both lungs. This phenomenon is due to the decrease in the inspiratory time with increased rates, decreasing the overall delivered volume during the later stages of the inspiratory cycle.

This study has a number of important limitations. Mainly, we have not tested this system on real patients during ventilator splitting. A real patient’s pulmonary mechanics might have subtle differences compared to the simplifications implicit in the test lung and computational model. For example, the model does not simulate the effect of alveolar recruitment, and therefore increasing PEEP at a given PIP never results in increased tidal volumes. Additionally, the current version of the VSRS excludes of the effects of differing patient pulmonary resistances, which we found to be a secondary effect compared to pulmonary compliances, but can be included in a new version of the computational model at a future date.

The VSRS is designed to initially have the patient on a single ventilator in order to determine their patient-specific pulmonary compliance and, in the case of volume-controlled ventilation, resistance. Once the patient is stabilized, they then can be moved to a split ventilator configuration using the VSRS for longer term ventilatory support with their now known patient-specific pulmonary characteristics entered into the app in order to choose the proper resistor size. Although not yet tested, in principle, the VSRS system could be deployed to support more than two patients on a given ventilator.

As with most recommendations related to splitting ventilators, the patients have to be fully sedated/paralyzed to eliminate them effecting each other’s respiratory rates and consequently, weaning of patients off of ventilator support cannot be performed while patients are sharing a ventilator. However, the goal behind ventilator splitting is to address periods of short supply, especially given reports of prolonged ventilator support times for COVID-19 patients.

Reserved for dire situations, ventilator splitting is complex, and introduces many safety concerns related to the lack of control over individual patient’s respiratory support, some of which are alleviated by the VSRS. By precomputing the hundreds of millions of different possible combinations of ventilator settings and patient-specific characteristics and by taking advantage of simple 3D printable geometries, the VSRS can be rapidly deployed at minimal cost wherever the need for ventilators surpasses their supply.

## Methods

### Study design

The project was designed to maximize the potential use cases for the VSRS, recognizing flexibility, ease of use, and rapid deployment as foundational pillars of a successful system to be used in dire situations. Consequently, the 3D printed design for the splitter and resistor was chosen to emphasize simplicity of use and ease of manufacturing to fit standard ventilator tubing. Benchtop testing of the VSRS was performed to test its applicability to both standard ICU ventilators and anesthesia machines, both of which could be required during times of ventilator shortages. Additionally, the benchtop testing provided the data necessary to calibrate the computational model. The computational model was used to precompute predicted tidal volumes and pressures for the large range of clinically relevant ventilator configurations and patient characteristics which clinicians might encounter when performing ventilator splitting and this was packaged into an easy to use mobile app for rapid clinical decision making support.

### Splitter and resistor design and manufacturing

The ventilator splitter and resistors were designed using SolidWorks (Dassault Systèmes, Vélizy-Villacoublay, France). It is designed to fit standard 22 mm tubing (Figure 1). The system consists of two primary components: the splitter and the resistor. Both components are manufactured from a commercially available, photopolymer via stereolithography (SLA). Both components are fully solid, continuous parts. The photopolymer is marketed as “Durable Resin” by Formlabs Inc. (Somerville, MA). All devices were printed on Form 2 printers manufactured by Formlabs Inc. All prints were done at restor3d (Durham, NC) following their internal quality system for resin-based 3D printing. The system underwent biocompatibility testing per FDA recommendation including ISO 18562 Part 2 (PM2.5/PM10 particulates) and Part 3 (VOCs) and was found to be biocompatible for the intended application.

### Benchtop test circuit set up

Experiments were primarily performed using a GE Aisys CS2 (GE Healthcare, Chicago, Illinois) anesthesia care station ventilator, with some experiments duplicated using a Covidien PB840 intensive care ventilator, to evaluate for differences in performance between OR and ICU ventilators with manipulation of circuit resistance and respiratory compliance. For added circuit resistance experiments, 3D printed resistors with round orifices were tested, one at a time, in each inspiratory circuit just distal to the circuit splitter. Ventilator and circuit components for both setups are presented, sequentially from ventilator inspiratory valve to expiratory valve, in Figure 1. “Proximal” refers to closer to the ventilator and “distal” refers to closer to the test lung (Linear Test Lung, Ingmar Medical, Pittsburgh, PA) side of a circuit. Care station automated self-tests were performed prior to testing on both single or split circuit setups (tubing fully extended). Some experiments were repeated with both single and split 6 ft. vs. 12 ft. tubing lengths. Bacterial/viral filters were placed on both inspiratory and expiratory limbs of the ventilator. One-way directional valves were then placed on both inspiratory and expiratory filters (Figure 1).

Pressure control mode was used with P_insp_ 20 cmH_2_O, RR 20 breaths/min, I:E ratio 1:2, and PEEP 5 cmH_2_O. Respiratory rates of 15 and 30 were also tested, with adjustment of I:E ratio to 1:1.5 during RR 30 testing, to maintain a more consistent (and clinically relevant) inspiratory time. Each linear test lung was tested with both “low” and “medium” compliance settings (manufacturer specifications: 10 and 30 mL/cmH_2_O, respectively). Ventilator parameters recorded were: peak inspiratory pressure (cmH_2_O), mean pressure (cmH_2_O), tidal volume (mL), and dynamic compliance (mL/cmH_2_O). Individual test lung circuit parameters recorded (each circuit, A and B) were: distal circuit maximum and trough pressure (mmHg), using a dry disposable pressure transducer (Transpac IV, ICU Medical, San Clemente, CA) connected directly to the gas sample port of each distal circuit filter. Pressures were recorded using a GE Carescape Monitor. Each distal circuit tidal volume was measured using in-line volume monitors (Ohmeda 6800 Volume Monitor, Bird Products, Palm Springs, CA).

### Reduced order ventilator computational model

A pressure-controlled and volume-controlled ventilator were considered for the computational model. A range of Reynolds numbers were simulated as a function of the ventilator settings, with higher Reynolds numbers approaching 4000. Air flow from the ventilator source to the patient was modeled using pipe flow dynamics in a gas network. The gas flow through the pipes is governed by the laws of mass, momentum and energy from which the pressure, velocity, density and temperature of the gas volume were solved.

The mass conservation relates the mass flow rates to the pressure and temperature of the gas volume by the following relationship[15]:

$$\frac{\partial \text{M}}{\partial \text{p}}\cdot \frac{{\text{dp}}_{\text{I}}}{\text{dt}}+\frac{\partial \text{M}}{\partial \text{T}}\cdot \frac{{\text{dT}}_{\text{I}}}{\text{dt}}={\dot{\text{m}}}_{\text{A}}+{\dot{\text{m}}}_{\text{B}}$$

were $\frac{\partial \text{M}}{\partial \text{p}}$ is the partial derivative of the mass of the gas flow with respect to pressure at constant temperature and volume. $\frac{\partial \text{M}}{\partial \text{T}}$ is the partial derivative of the mass of the gas volume with respect to temperature at constant pressure and volume, P_I_ is the pressure of the gas volume, T_I_ is the temperature and t is time. M is the mas of gas entering the device. ${\dot{\text{m}}}_{\text{A}}$ and ${\dot{\text{m}}}_{\text{B}}$ are the mass flow rates to the patients A and B, respectively.

Energy conservation is given by the following relationship[16]:

$$\frac{\partial \text{U}}{\partial \text{p}}\cdot \frac{{\text{dp}}_{\text{I}}}{\text{dt}}+\frac{\partial \text{U}}{\partial \text{T}}\cdot \frac{{\text{dT}}_{\text{I}}}{\text{dt}}=\Phi_{\text{A}}+\Phi_{\text{B}}+{\text{Q}}_{\text{H}}$$

$\frac{\partial \text{U}}{\partial \text{p}}$ is the partial derivative of the internal energy of the control volume with respect to pressure at constant temperature and volume. $\frac{\partial \text{U}}{\partial \text{T}}$ is the partial derivative of the internal energy of the control volume with respect to temperature at constant pressure and volume. Φ_A_ and Φ_B_ are the energy flow rates to the patients. Q_H_ represents the energy flow rate from the pipe wall.

Pressure loses due to viscous friction are given by the momentum balance relationship[17]:

$${\text{p}}_{\text{A}}-{\text{p}}_{\text{I}}={\left(\frac{{\dot{\text{m}}}_{\text{A}}}{\text{S}}\right)}^{2}\cdot \left(\frac{1}{\rho_{\text{I}}}-\frac{1}{\rho_{\text{A}}}\right)+\Delta {\text{p}}_{\text{AI}}$$

$${\text{p}}_{\text{B}}-{\text{p}}_{\text{I}}={\left(\frac{{\dot{\text{m}}}_{\text{B}}}{\text{S}}\right)}^{2}\cdot \left(\frac{1}{\rho_{\text{I}}}-\frac{1}{\rho_{\text{B}}}\right)+\Delta {\text{p}}_{\text{BI}}$$

p_A_ and p_B_ are the pressures at the inlet and the outlet of the pipe respectively. ρ_A_ and ρ_B_ represent densities at the inlet and outlet of the pipe. S is the cross sectional area and Δp_AI_ and Δp_BI_ are the pressure loses due to friction.

The two patients are connected to each other by way of a junction, with a resistor connected distal to one of the branches to enable controlling the differential flow. The lungs are modeled as a Hookean spring (modeling the inverse of the compliance of the lungs) and a viscous dashpot (modeling the resistance of the upper respiratory tract) in parallel. This lung model presented in this study is consistent with other studies that have used a resistor - capacitor model to represent the lungs[17]. The simulations were performed using MathWork’s Simscape (Simulink v4.8) Foundational Blocks.

The ventilator is simulated by a pulse wave form generator with a period corresponding to the respiratory rate and inspiratory time, with a maximum value corresponding to the PIP and minimum value corresponding to the PEEP.

To explore the relevant possible range of ventilator settings, endotracheal tube diameters, and lung compliance values (Table 2), over 200 million different simulations were performed, consuming over 800,000 computer hours. In order for the system to reach steady state, at least 5 breath cycles were simulated for each set of parameters. Sensitivity tests were conducted to determine the important parameters to simulate and the step size was selected such that a change in tidal volume of less than 5% occurred for a given step size.

Simulations were performed by combining the resources of the Duke Compute Cluster and Microsoft’s Azure cloud platform. On Azure, 24,000 compute cores (400 HB60rs nodes, each with 240 GB of RAM and 60 cores) were utilized to allow for the completion of all simulations in one weekend. Postprocessing was done on node, reducing the 100 TBs of time series data to a ~10 GB data table summarizing all of the computational results.

## Supplementary Material

Supplement

Supplement

## Figures and Tables

**Figure 1:** Splitter and resistor design, and circuit configuration Overall design (1A) and final product (1B) for the splitter and resistor system. Benchtop circuit set up is shown in figure 1C. The circuit incorporated a number of one-way valves at the inspiratory and expiratory limbs of both patients to limit mixing. Furthermore, viral/bacterial filters were placed at locations of possible cross contamination.

**Figure 2:** Changes in delivered tidal volumes during ventilator splitting to lungs with different compliances Changes in delivered tidal volumes to test lung A and test lung B under different respiratory rates for different compliance configurations. Delivered volumes were similar to both lungs when they both had similar compliances (both low or both medium). As respiratory rate increases, overall delivered volumes decreased. The differential tidal volume delivered under different compliances decreases as respiratory rates increase from 15 to 30 breath per minute.

**Figure 3:** Changes in delivered tidal volumes during ventilator splitting using different resistor luminal apertures Changes in delivered tidal volumes to test lung A and test lung B for different compliance settings without a resistor vs differently sized resistors. Delivered tidal volumes to the lung with the smaller resistors is decreased compared to cases with the larger resistors or without a resistor. For this set up, the 3.5 mm resistor creates similar delivered volumes to the low and medium compliance lungs.

**Figure 4:** Comparison of the numerical results to the benchtop experimental data Comparison of the simulation results (dotted lines) with the benchtop results (large circles) for patient A (medium compliance) and patient B (low compliance). Both are in agreement over a range of resistor sizes and demonstrate similar delivered tidal volumes to both patients using a 3.5 mm resistor.

**Figure 5:** Model output waveforms for pressure, flow rate, and tidal volume Example model output for simulated patient A (compliance of 30 ml/cmH_2_O) and simulated patient B (compliance of 75 ml/cmH_2_O) using a 4 mm resistor. While Patient A experiences the peak inspiratory pressure set by the ventilator, the presence of the resistor results in decreased peak pressures to Patient B.

**Figure 6:** Comparing pressure-controlled and volume-controlled ventilation in the split ventilator configuration Computational model output of tidal volume (top row) and peak inspiratory pressure (bottom row) for different resistor sizes under pressure-controlled ventilation (left column) and volume-controlled ventilation (right column). Pressure-controlled ventilation results in a decoupling of the two patients, where patient A’s tidal volumes and pressures are not affected by patient B’s circuit. This is not the case for volume-controlled ventilation, which can result in dangerously high tidal volumes and pressures for patient A as a result of increases in resistance in patient B’s circuit and vice versa.

**Figure 7:** Multiple dimensions of nonlinearity for predicted tidal volumes The predicted volumes for a 8.5 mm endotracheal tube compared to a 6 mm endotracheal tube as a function of pulmonary compliance and respiratory rate with other ventilator settings held constant (PIP of 28, PEEP of 8, I:E of 1).

**Figure 8:** Illustration of the results from computational model’s parameter sweep Upper left: The average tidal volume without a resistor for all simulations as a function of PIP, PEEP, and I:E. Upper right: Average tidal volume without a resistor for all simulations originating from the black square on the upper left figure (PIP of 28, PEEP of 8, I:E of 1) as a function of RR, compliances, and endotracheal tube diameters. Lower panels: the calculated tidal volumes (left side) and maximum delivered pressures (right side) for patients with ventilator settings and pulmonary characteristics A and B as depicted from the Upper Right panel as a function of resistor characteristics.
