# Supplementary figures and images for: Rapid Ventilator Splitting During COVID-19 Pandemic Using 3D Printed Devices and Numerical Modeling of 200 Million Patient Specific Air Flow Scenarios
Source: Res Sq. 2020 Aug 12:rs.3.rs-48165. Preprint. [Version 1] doi: 10.21203/rs.3.rs-48165/v1 (PMC7430577; doi:10.21203/rs.3.rs-48165/v1)

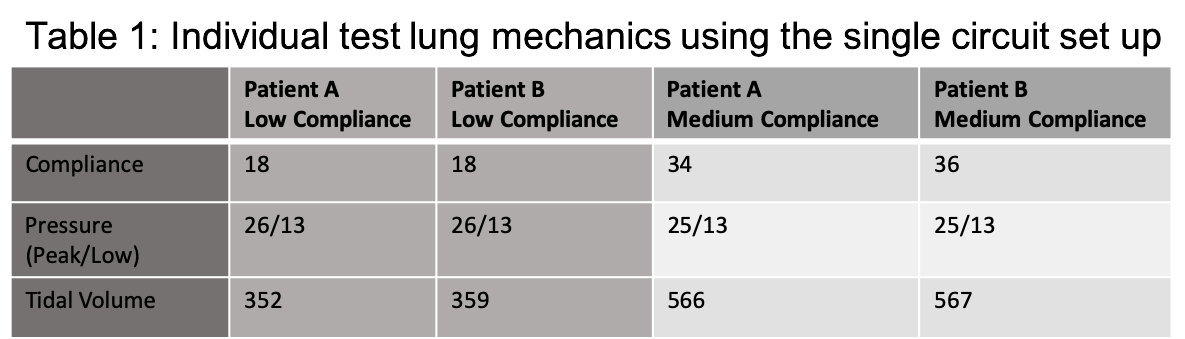

Supplement: Supplement [file Table1.png]

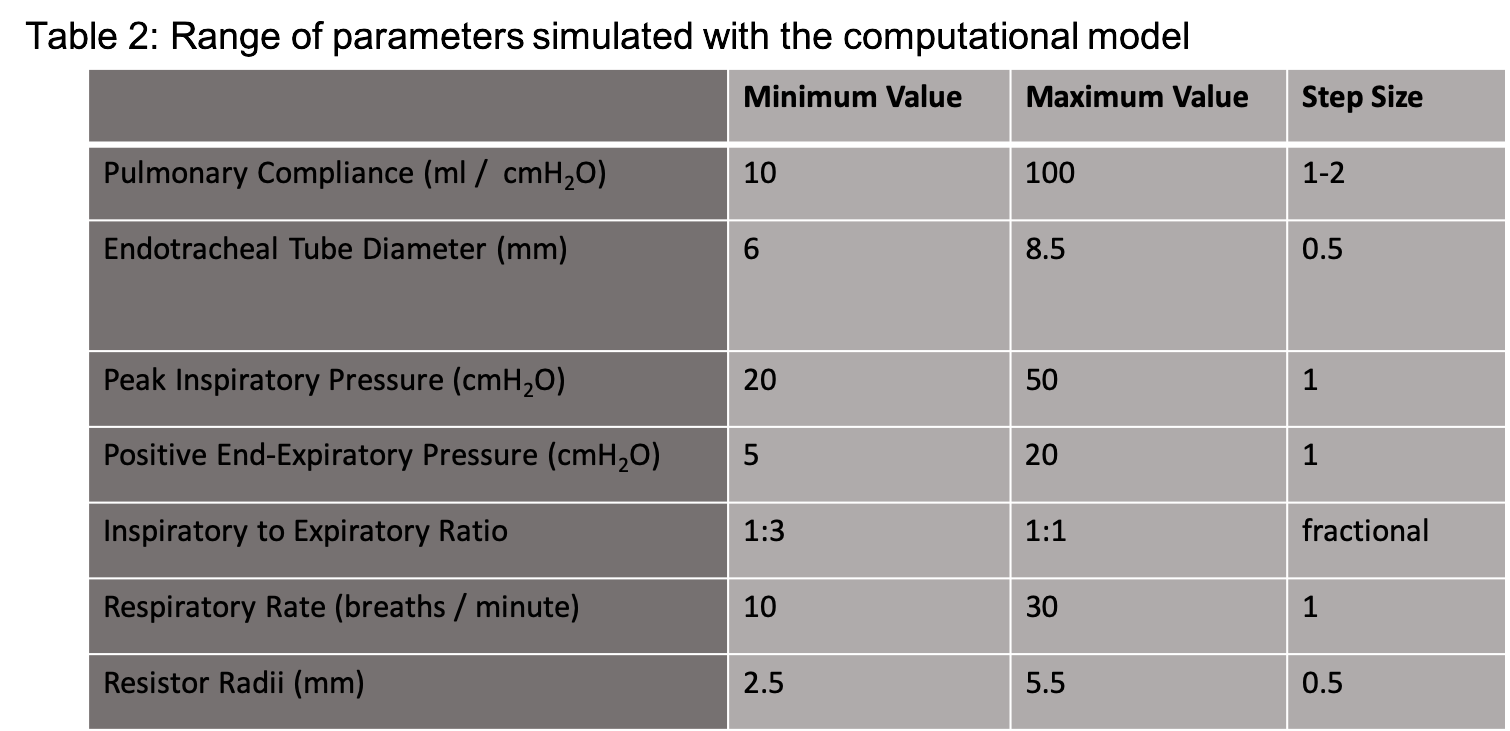

Supplement: Supplement [file table2.png]
